# Estimates of the Number of People Living with HIV in Italy

**DOI:** 10.1155/2014/209619

**Published:** 2014-07-17

**Authors:** Laura Camoni, Vincenza Regine, Karen Stanecki, Maria Cristina Salfa, Mariangela Raimondo, Barbara Suligoi

**Affiliations:** ^1^Centro Operativo AIDS, Dipartimento di Malattie Infettive Parassitarie e Immunomediate, Istituto Superiore di Sanità, Viale Regina Elena 299, 00161 Rome, Italy; ^2^Joint United Nations Programme on HIV/AIDS (UNAIDS), 20, Avenue Appia, CH-1211 Geneva 27, Switzerland

## Abstract

*Objective*. To estimate the HIV prevalence and the number of people living with HIV (PLHIV) in Italy with a projection for 2020. *Methods*. Two methods elaborated by Joint United Nations Programme on HIV/AIDS (UNAIDS) were used: Estimate and Projection Package and Spectrum. *Results*. A total of 123,000 (115,000–145,000) individuals aged 15 or more were estimated to be living with HIV in Italy at the end of 2012 and the estimated HIV prevalence was 0.28 (0.24–0.32) per 100 residents aged 15 or more. In 2012, the estimated number of new HIV infections among adults was 3,000 (2,700–4,000), and the number of adults in need for ART was 93,000 (80,000–110,000). The projection estimates that 130,000 (110,000–150,000) adults will live with HIV/AIDS in 2020 in Italy. *Conclusion*. Estimates of PLHIV in Italy stress the high number of PLHIV in need of care and treatment, as well as the need for more information and prevention campaigns.

## 1. Introduction

The HIV epidemic continues to affect the European countries with different magnitude and dynamics. Italy, with about 60 million of inhabitants and about 4 thousand new diagnoses of HIV infection annually, contributes significantly to outline the profile of the HIV epidemic in Europe. Several types of data sources contribute to draw the dynamics of the HIV epidemic in Italy: the surveillance of AIDS cases that provide information on individuals with AIDS and the surveillance of new HIV diagnoses that provide information on people only newly diagnosed with HIV; both the surveillances are mandatory and have a national coverage. Also, there are several prevalence studies on specific population groups which provide a snapshot on the spread of the infection in subpopulations at different levels of risk of infection (such as pregnant women, blood donors, injecting drug users (IDUs), men who have sex with men (MSM), and female sex workers (FSW)).

Data from the above mentioned two surveillance systems show that the annual trend of both incident AIDS cases and new HIV diagnoses in Italy has leveled off in the last decade and the number of people living with HIV has increased since the introduction of ART in 1996 [1].

In 2009, the number of people living with HIV (PLHIV) in Italy was calculated using data obtained from the national surveillance of AIDS cases and the surveillance of new HIV diagnoses. Specifically we used the number of new HIV diagnoses, the number of new AIDS cases, and the number of AIDS deaths reported by physicians. According to this calculation, in 2009, there were approximately 140,000 PLHIV in Italy [2].

The aim of the present study is to estimate the number of PLHIV in Italy in 2012, using a method elaborated by the Joint United Nations Programme on HIV/AIDS (UNAIDS) [3]. A projection for 2020 was also provided.

## 2. Methods

To estimate HIV prevalence, we used a tool designed by UNAIDS: Estimation and Projection Package (EPP)/Spectrum (version 4.66) [3, 4].

The Spectrum and Estimation and Projection Package (EPP) programs are used to estimate key HIV indicators based on HIV surveillance and surveys, programme statistics, and epidemic patterns. These indicators include the number of people living with HIV, new infections, AIDS deaths, AIDS orphans, the number of adults and children needing treatment, the need for preventing mother to child transmission (PMTCT), and the impact of antiretroviral treatment on survival. Countries have been using Spectrum and EPP with training and guidance from UNAIDS since 2003 [5–8].

The projections start with an estimate and projection of adult incidence, which is combined with information on the age and sex distribution of incidence and progression to death to estimate the number of new adult infections by age and sex.

New infections progress over time to lower CD4 counts and are subject to AIDS-related mortality. Those who receive first-line and/or second-line antiretroviral treatment (ART) experience extended survival. People at any stage are also subject to non-AIDS mortality at the same rates as those who are not infected [7].

This method can be applied both to generalized and concentrated epidemics. Italy fully meets the definition of “concentrated epidemic” of UNAIDS; that is, HIV prevalence among pregnant women is less than 1% and over 5% in at least one subpopulation such as IDUs and MSM in Italy [9, 10].

EPP uses data obtained from both surveillance systems and surveys conducted in subpopulations; it requires data on subpopulation size and at least three HIV prevalence data points for every subpopulation included. For this analysis, we considered that the following subpopulations are defined to be mutually exclusive: IDUs, MSM, FSW, multipartner heterosexual males (MHET), and male and female remaining population (males and females not included in any of the previous subpopulations, such as non-IDU or nonmultipartner male heterosexuals and non-IDU or non-FSW females, as well as IDUs, MSM, FSW, and MHET after the mean time of permanence in their own subpopulation). Foreigners are not considered separately.

Spectrum is a modular program that uses data obtained from EPP and applies demographic corrections, examines effects and projections of the HIV epidemic based on estimated prevalence, and provides results stratified by age group and calendar year. Among the modules included in Spectrum, we used the AIDS Impact Model (AIM), and we selected the parameters for developed countries. Spectrum requires inputting data on ART, specifically on number of HIV positive pregnant women on ART, on number of children on ART, and on number of adults on ART. Moreover, Spectrum takes into account mortality data and the survival expected for people on ART. This model produces national estimates of PLHIV stratified in two age groups: young people (15–24 years old) and adults (people older than 15 years). Using uncertainty analysis function [11], Spectrum generates the range for the estimate obtained by applying the plausibility bounds of 95% as measure of its accuracy around the median (lower and upper bound estimates) and 2.5–97.5% percentiles of the resulting curves.

### 2.1. Data Required

*Estimated Size of Subpopulations*

- IDUs: we used annual data obtained from the Annual Report on Drug Use in Italy prepared by the Ministry of Health, available from 1991 to 2011 [12]. These data report the number of both injecting and noninjecting drug users attending Public Drug Treatment Centers (PDTC) at national level; all 550 PDTC operating in Italy participate in this network. To obtain the number of IDUs, we used the results of a study recently conducted in Italy [13, 14], which shows that IDUs account for 75% of individuals attending PDTC. Therefore, we calculated the number of IDUs in Italy as 75% of the total number of drug users reported by any given year in the Annual Report on Drug Use.
- MSM: we used published data from a recent survey of the Italian National Institute of Statistics. This survey on “Discrimination based on gender, sexual orientation and ethnicity” was carried out in 2011 among a representative sample of 7,725 adults (18–74 years) and reports the percentage of males defining themselves as MSM [15].
- FSW: we used the estimates obtained from two studies conducted in Italy: “Parsec” from 1995 to 1999 and “Europap” from 1998 to 2000 [16]. These estimates include only females; no data on male sex workers are available in Italy, mainly because this group is small and hard to reach [17].
- MHET: we used the minimum number of MHET that we can approximate with the number of clients of FSW. This calculation was based on the average annual number of male clients reported by FSW, ranging from 20 to 30 clients per FSW (personal communication, Pia Covre, 2008).

For every subpopulation, we included the gender distribution and the mean time of permanence in the group. Specifically, based on the results of the above mentioned surveys and studies, we assigned the following: IDUs are composed of 90% males and remain in this group for 12 years; MSM are composed of 100% males and remain in this group for 40 years; FSW are all females and remain in this group for 10 years; MHET are composed of 100% males and remain in this group for 10 years.

#### 2.1.1. HIV Prevalence Based on Surveillance Data

HIV prevalence based on surveillance data was obtained from the following sources:

- the Annual Report on Drug Use in Italy, which collects data on drug users attending PDTC at national level; annual HIV prevalence is available between 1991 and 2011;
- the HIV surveillance system, which collects data on new HIV diagnoses; annual HIV prevalence is obtained by summing the number of new HIV cases reported every year between 1985 and 2011 and then subtracting the number of AIDS deaths;
- the STD sentinel surveillance system, which collects information on STD cases reported by 12 public STD clinics located in major cities throughout Italy; every individual with a confirmed STD is asked to undergo HIV testing; HIV prevalence is obtained from the results of this screening.

The HIV prevalence data was available for the following subpopulations.

- IDUs: we used the HIV prevalence reported by the Annual Report on Drug Use in Italy, per year, from 1991 to 2011 [13, 14].
- MSM: we used the HIV prevalence among non-IDU MSM reported both by the HIV surveillance system and by the STD sentinel surveillance system, per year, from 1991 to 2011 [1, 18].
- FSW: we defined as FSW women with more than five sexual partners a year. We used the HIV prevalence among non-IDU women who had more than five sexual partners in the last year, reported by the STD sentinel surveillance system, per year, from 1991 to 2011 [18].
- MHET: we used the prevalence among non-IDU heterosexual men who had more than five sexual partners in the last year, reported both by the HIV surveillance system and by the STD sentinel surveillance system, per year, from 1991 to 2011.

#### 2.1.2. HIV Prevalence Based on Surveys

We used data obtained from surveys conducted among IDUs, MSM, and FSW.

- IDUs: in 2005 and 2007, two surveys were conducted in a randomized sample of drug users attending PDTC. These studies found an HIV prevalence of 14.4% in 2005 and 19.0% in 2007 [13, 14].
- MSM: three surveys were conducted in this population: the MODI DI. survey in 2005 [19], which showed a prevalence of 4%; the SIALON study in 2008 [20], which showed a prevalence of 11.9%; and the EMIS study in 2010 [21], which showed a prevalence of 9.6%.
- FSW: five surveys were conducted in different Italian cities showing the following HIV prevalence rates: Verona 1.8% [22], Bologna 1.6% [23], Brescia 2.5% [24], Rome 6.0% [25], and Palermo, 1.8% [26].

#### 2.1.3. ART Data

We used the following sources for information on ART.

- Distribution of HIV+ pregnant women by treatment regimen and number of children receiving ART: we used data from the “Italian Group on Surveillance of Antiretroviral Treatment in Pregnancy” coordinated by the National Institute of Health [27], from 1996 to 2011.
- Number of adults receiving ART: we estimated the number of individuals receiving ART using data obtained from a survey conducted in Italy in 2012 with the collaboration of all the Italian Infectious Diseases clinics [28].
- Eligibility criteria for ART in adults and children: we used the last Italian Guidelines on ART [29] and the last Italian Guidelines that considers the individuals with CD4 < 350 cells/mL as eligible for treatment.

## 3. Results

A total of 123,000 (115,000–145,000) individuals aged 15 or more were estimated to be living with HIV/AIDS in Italy at the end of 2012: 110,000 (94,000–130,000) males and 13,000 (11,000–15,000) females (Table 1).

The estimated HIV prevalence among adults in Italy in 2012 was 0.28 (0.24–0.32) per 100 residents aged 15 or more; the prevalence among males and females aged 15–24 was 0.10 (0.08–0.13) per 100 male residents and 0.02 (0.02–0.03) per 100 female residents of the same age group, respectively.

In 2012, the estimated number of new HIV infections among adults was 3,000 (2,700–4,000); 700 (600–800) new infections were estimated among males aged 15–24 and 90 (70–100) among females aged 15–24.

The estimated number of adults with AIDS who died in 2012 was 1,500 (1,200–1,700) and the number of adults receiving ART was 93,000 (80,000–110,000).

**Figure 1:**
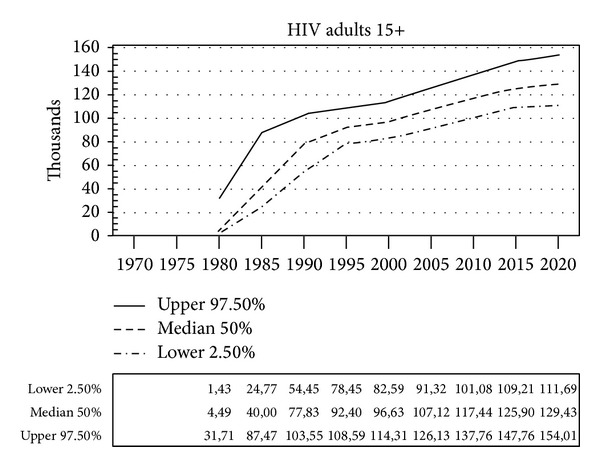
Number of adults (15 years or more) living with HIV in Italy, 1980–2020 (Spectrum projection).

The projection estimates that 130,000 (110,000–150,000) adults will live with HIV in 2020 in Italy (Figure 1). Figure 1 describes a 20-fold rapid increase in the number of adults with HIV/AIDS between 1980 and 1995. The estimated number of PLHIV increases by 30% between 1995 and 2012 and by 7.5% between 2012 and 2020.

In 2012, the distribution of PLHIV by subpopulation was as follows: 38% MSM, 16% IDUs, 7% FSW, 7% MHET, and 32% remaining population.

**Figure 2:**
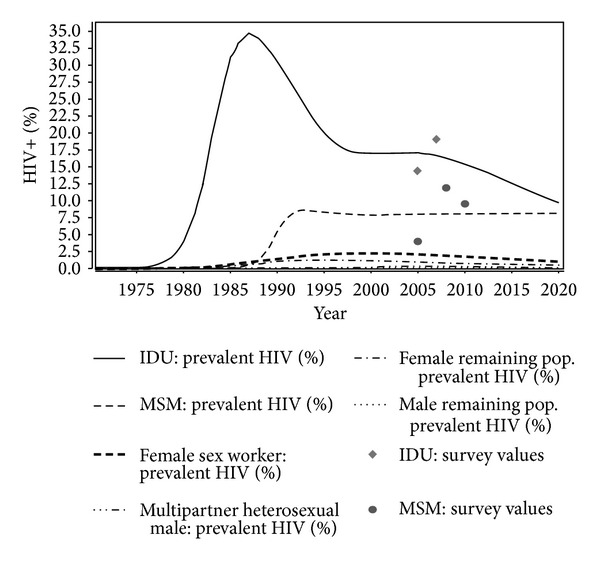
Distribution of people living with HIV in Italy, by subpopulation (EPP projection).

The EPP module depicts the trend of HIV prevalence in every subpopulation of interest, from the estimated beginning of the epidemic from 1975 to 2020 (Figure 2). The model shows a relevant rise in HIV prevalence among IDUs in the early 80s (from 3.8% in 1980 to 34.6% in 1987) and among MSM in the early 90s (from 1.1% in 1988 to 8.6% in 1993). After 1987, the prevalence among IDUs shows a sharp decrease, whereas the prevalence among MSM remains relatively stable after 1993. Much lower prevalence rates are observed overall among FSW and MHET, with a slight prevalence increase in both subpopulations until 1999 (from 0.1% in 1982 to 2.2% in 1999 and from 0.1% in 1982 to 1.1% in 1999, resp.) and a low decrease afterwards. Also, in the male and female remaining population, an increase in prevalence is observed until 1985 (0.007% and 0.004%, resp., in 1985); however, after 1985, prevalence in the female remaining population increases until 2000 and then remains stable (0.04% in 2020) whereas that in the male remaining population increases gradually (from 0.007% in 1985 to 0.1% in 2020). In 2012, the estimated prevalence among IDUs was 14.9%, among MSM 8.0%, among FSW 1.6%, among MHET 0.6%, in the male remaining population 0.13%, and in the female remaining population 0.05%.

## 4. Discussion

This analysis presents the first estimate of PLHIV in Italy which is approximately of 123,000 adults in 2012 with a prevalence of 0.28 per 100 adults. This estimated HIV prevalence is slightly lower than that reported for Italy (0.30%) in the UNAIDS Global Report in 2012 using the same method, because we used the 2013 updated version of the EPP/Spectrum package that tends to reduce the prevalence estimates compared to previous versions due to the introduction of additional parameters. Moreover, this estimate is lower than that reported in 2009 [2] because the 2009 estimate did not include non-AIDS mortality.

The overall HIV prevalence trend shows a permanent increase of PLHIV over time, primarily because of the beneficial effects of antiretroviral treatment (longer survival of PLHIV and decrease in the number of deaths correlated with AIDS) not being compensated by a decrease in the number of new infections [1].

The results of the model indicate that the HIV epidemic in Italy occurred with the main contribution of two subpopulations: IDUs in the early phase of the epidemic in the 80s and MSM since the 90s. The estimated number of PLHIV infected through injecting drugs was very high at the beginning of the epidemic but had halved between 1985 and 2012; this result is in agreement with data reported to the HIV surveillance system showing that the highest number of new HIV cases was diagnosed in the late 80s and about 80% of them were IDUs, whereas the proportion of IDUs among new HIV diagnoses was 5% in 2012 [1]. The decline of IDUs living with HIV which is predicted by the model can be attributed to the following factors: the progressive reduction of the number of individuals who inject drugs, the mortality due to AIDS and other blood-transmitted infections, the decline in the proportion of susceptible HIV-uninfected drug users over time, and the efficacy of prevention campaigns and harm reduction programs conducted in all Italian PDTC which started in the late 80s [12] and that did not include free distribution of needles. The stabilization of the estimated prevalence trend between 1995 and 2007 can be partially due to the introduction of ART in 1996 leading to an improved survival.

The beginning of the epidemic among MSM is estimated about a decade later than that among IDUs, at a minor level in terms of prevalence. In fact, the annual prevalence rates among MSM were always lower compared to IDUs and the peak prevalence among MSM in 1993 was fourfold lower compared to that among IDUs in 1987. However, these differences are greatly reduced when taking into account the absolute number of PLHIV in the two subpopulations. The peak number of PLHIV among MSM in 1993 was similar to that reached by IDUs a few years earlier in 1987; after 1990, the annual number of MSM living with HIV always exceeded that of IDUs and remained relatively stable over time whereas that of IDUs showed a marked decrease. The rather stable prevalence trend predicted by the model after 1990 among MSM may imply that, in the last two decades, the incidence has been fairly constant and the mortality was low or the incidence increased with minor changes in mortality rates.

Estimated HIV prevalence trends among MHET and the male remaining population appears to be negligible compared to that among IDUs or MSM, but trends in absolute numbers provide more detailed information. In the late 90s, the estimated number of MHET living with HIV increased and was very close to that of IDUs living with HIV in the same period; after 2000, the reduction of MHET living with HIV was offset by the slow and progressive increase of PLHIV in the male remaining population. These estimates suggest that the HIV epidemic in Italy received a significant contribution from males in various subpopulations (IDUs, MSM, MHET, and the male remaining population) and that, after 2000, most of PLHIV was constituted by individuals infected through sexual contact.

Different from other approaches [30], the estimates of the total number of PLHIV obtained with EPP/Spectrum package do not specify the proportion of undiagnosed cases, which is an important piece of information for planning HIV screening strategies and promotion of HIV testing in specific risk groups.

Some limitations of the results obtained must be underlined. First, the number of about 1500 AIDS deaths estimated for 2012 appears to be too high compared to the approximately 700 AIDS deaths reported by the mortality database of the Italian Institute of Statistics [1]. Second, the estimated 10% proportion of adult females living with HIV/AIDS in 2012 seems too small when considering that the proportion of females reported to both the HIV surveillance system and the AIDS surveillance system was never lower than 20% [1]. Third, the present estimates do not specify the proportion of undiagnosed cases, which is an important piece of information for planning HIV screening strategies and promotion of HIV testing in specific risk groups.

A possible explanation for the above mentioned limitations is that the EPP/Spectrum package was primarily designed for use in countries with generalized epidemics where large subpopulations at risk and high prevalence rates are observed. When applied in countries with concentrated epidemics, as in Italy, a certain level of uncertainty on subpopulation size or a poor representativeness of prevalence surveys can lead to some inaccuracy in estimates, and small fluctuations in the values inputted for these parameters can generate fairly important differences in estimate. The results obtained highly depend on the type of data used; for example, the low estimated number of females living with HIV/AIDS may be associated with the small amount of information on females derived from datasets (including mainly males, such as MSM, IDU, and MHET) used to feed the model. Nevertheless, the 2013 updated version of EPP/Spectrum package has already been improved compared to previous versions by taking into account parameters that are of relevance for epidemics in industrialized countries, such as the high proportion of individuals on ART and the low rate of vertical HIV transmission. Although no corrections can be applied to the model for specific parameters, the overall depiction of the epidemic and short-term projections are valuable and are in agreement with surveillance findings.

## 5. Conclusions

The EPP/Spectrum package produces helpful short- and long-term estimates, is user-friendly, requires no specific statistical experience, and implies simple data input and low expertise to manage the process.

Estimates of PLHIV in Italy stress the high number of PLHIV in need of care and treatment, as well as the need for more information and prevention campaigns, to be conducted primarily among males. These results are essential to understand and interpret the spread of HIV/AIDS in Italy, to make comparisons with other estimation methods and results obtained in other countries, and to provide an epidemiological baseline for the development of a new national AIDS program for the next years.

## Figures and Tables

**Table 1:** EPP/Spectrum estimates for Italy, 2012.

	Estimate	Lower bound estimate–higher bound estimate
Number of PLHIV: adults aged 15+	123,000	(115,000–145,000)
Number of PLHIV: males aged 15+	110,000	(94,000–130,000)
Number of PLHIV: females aged 15+	13,000	(11,000–15,000)
Number of PLHIV: males aged 15–24	3,200	(3,000–3,600)
Number of PLHIV: females aged 15–24	600	(500–700)
Number of new HIV infections: adults aged 15+	3,000	(2,700–4,000)
Number of new HIV infections: males aged 15–24	700	(600–800)
Number of new HIV infections: females aged 15–24	90	(70–100)
Number of AIDS deaths: adults aged 15+	1,500	(1,200–1,700)
Number of individuals receiving ART: adults aged 15+	93,000	(80,000–110,000)
